# Endocannabinoids and Fear-Related Behavior in Mice Selectively Bred for High or Low Alcohol Preference

**DOI:** 10.3390/brainsci9100254

**Published:** 2019-09-26

**Authors:** Aaron M. Kirchhoff, Eric L. Barker, Julia A. Chester

**Affiliations:** 1 Department of Psychological Sciences, Purdue University, West Lafayette, IN 47907, USA; akirchho@scripps.edu; 2 Department of Medicinal Chemistry and Molecular Pharmacology, Purdue University, West Lafayette, IN 47907, USA; barkerel@purdue.edu

**Keywords:** alcohol preference, amygdala, anandamide, estrous, endocannabinoid, fear, hippocampus, mice, prefrontal cortex, post-traumatic stress disorder

## Abstract

Alcohol use disorders (AUDs) have a high incidence of co-morbidity with stress-related psychopathologies, such as post-traumatic stress disorder (PTSD). Genetic and pharmacological studies support a prominent role for the endocannabinoid system (ECS) in modulating stress-related behaviors relevant to AUDs and PTSD. Mouse lines selectively bred for high (HAP) and low (LAP) alcohol preference show reproducible differences in fear-potentiated startle (FPS), a model for PTSD-related behavior. The first experiment in this study assessed levels of the endocannabinoids, anandamide (AEA) and *sn*-2 arachidonylglycerol (2-AG), in the prefrontal cortex (PFC), amygdala (AMG), and hippocampus (HIP) of male and female HAP1 and LAP1 mice following the expression of FPS to determine whether ECS responses to conditioned-fear stress (FPS) were correlated with genetic propensity toward high or low alcohol preference. The second experiment examined effects of a cannabinoid receptor type 1 agonist (CP55940) and antagonist (rimonabant) on the expression of FPS in HAP1 and LAP1 male and female mice. The estrous cycle of females was monitored throughout the experiments to determine if the expression of FPS differed by stage of the cycle. FPS was greater in male and female HAP1 than LAP1 mice, as previously reported. In both experiments, LAP1 females in diestrus displayed greater FPS than LAP1 females in metestrus and estrus. In the AMG and HIP, AEA levels were greater in male fear-conditioned HAP1 mice than LAP1 mice. There were no line or sex differences in effects of CP55940 or rimonabant on the expression of FPS. However, surprisingly, evidence for anxiogenic effects of prior treatment with CP55940 were seen in all mice during the third drug-free FPS test. These findings suggest that genetic differences in ECS function in response to fear-conditioning stress may underlie differences in FPS expression in HAP1 and LAP1 selected lines.

## 1. Introduction

Alcohol use disorders (AUDs) have a high incidence of co-morbidity with stress-related psychopathologies, such as post-traumatic stress disorder (PTSD) [1,2]. There is currently a critical need to identify appropriate prevention and treatment strategies for these disorders that produce a tremendous impact on the individual and society both in terms of human suffering and economics [3]. The risk for developing these co-morbid disorders is strongly influenced by interactions with genetic/biological and environmental factors, such as stress exposure. Genetic correlation studies have shown significant overlap in genes linked to the expression of both AUDs and PTSD [4]. Although stress has long been known to be a risk factor for AUDs and co-morbid stress-related psychopathologies [5], the mechanisms by which stress-gene-interactions promote vulnerability or resilience toward developing stress-related disorders is a complex research area. 

There have been significant research efforts in exploring the ECS as a promising biological target for pharmacotherapies to treat both PTSD and AUDs [6,7,8,9]. The two main endogenous cannabinoid signaling molecules are anandamide (AEA) and 2-arachidonoyl glycerol (2-AG), produced on demand from phospholipids, which, in turn, bind to the two following main receptor subtypes: Cannabinoid receptor type 1 (CB1) and 2 (CB2) [10,11,12]. Two hydrolytic enzymes, fatty-acid amide hydrolase (FAAH) and monoacylglycerol lipase (MAGL), are the primary catabolic enzymes responsible for the breakdown of AEA and 2-AG, respectively [13,14]. AEA and 2-AG bind to CB receptors to modulate tonic and phasic neuronal excitability and short and long-term synaptic plasticity [15,16]. Genetic studies in humans have identified functional mutations in the CB1 receptor gene (CNR1) and fatty acid amide hydrolase (FAAH) in individuals with PTSD [17], AUDs [18,19], and those with co-occurring PTSD and AUD [8]. Additionally, genetic and pharmacological studies in animal models support a prominent role for the ECS in modulating alcohol- and stress-related behaviors relevant to AUDs [20] and PTSD [21]. 

The ECS plays a central role in regulating the hypothalamic-pituitary-adrenal (HPA) axis response to, and recovery from, stress exposure as well as expression of anxiety and fear-related behaviors [22,23,24,25,26,27]. Stress exposure can cause a decrease in AEA levels [28,29], through mobilization of FAAH, and an increase in 2-AG levels [30], in brain regions such as amygdalar and prefrontal cortical areas, which are critical for the expression of anxiety and fear-related behaviors. Inhibition of FAAH through gene deletion [31] or pharmacological inhibition [28,29] can interfere with stress-induced reductions in AEA and the associated anxiety- and learned fear-like behaviors, an effect mediated through AEA-CB1 receptor interactions in amygdalar brain regions [32]. Gene knockout mice lacking the main 2-AG producing enzyme, diacylglycerol lipase α (DAGL-α), showed impairments in a variety of stress-related behaviors, including increased anxiety-related behaviors and impaired extinction of conditioned fear [33]. Augmenting 2-AG is associated with stress-resilience, whereas depletion of 2-AG or CB1 receptor antagonism produced susceptibility to stress [23]. 

A primary goal of translational research is to develop valid animal models which can isolate gene and environmental factors to help understand how these factors and their interactions promote vulnerability toward or resilience against stress-related disorders. In our research program, we use a unique and relevant animal model for genetic risk factors that may contribute to co-morbid AUD and PTSD, as follows: Mouse lines selectively bred for high (HAP) and low (LAP) alcohol preference. These lines show reproducible differences in fear- and neuroendocrine-related phenotypes in response to stress that are genetically correlated with selection for high or low alcohol preference. This animal model is a useful pre-clinical model to identify effective pharmacotherapeutic treatments for individuals with co-morbid disorders, particularly in individuals with a family history (genetic risk) of AUDs and/or PTSD. These treatments are currently in high demand due to the relatively low therapeutic effectiveness of current pharmacotherapies.

Conditioned fear-potentiated startle (FPS) is one model of cued fear-related behavior used to study associative learning processes that support the development and maintenance of PTSD symptomatology [34]. The FPS model has face, construct, and predictive validity [35,36,37,38], and therefore provides a valid and relevant methodology for identifying treatments for PTSD. We have done extensive work repeatedly demonstrating that HAP1 and HAP2 mouse lines show greater FPS than LAP1 and LAP2 lines [39,40,41]. As both independently selected lines showed the correlated response to selection, these findings indicate there are common overlapping genes influencing propensity for alcohol preference and FPS [42]. We previously reported that an EC uptake inhibitor (LY2183240) reduced the expression of FPS in HAP1/2, but not in LAP1 mice [43], suggesting genetic differences in ECS function between HAP1 and LAP1 mice. 

The current study sought to examine the ECS following the expression of FPS to determine whether ECS responses to conditioned-fear stress (FPS) were correlated with genetic propensity toward high or low alcohol preference. In the first experiment, we measured AEA and 2-AG in the prefrontal cortex (PFC), amygdala (AMG), and hippocampus (HIP) following a FPS test in HAP1 and LAP1 male and female mice. These brain regions were chosen because FAAH and the CB1 receptor are highly expressed in these regions, which are known to regulate alcohol-reward related behavior [44] and fear- and anxiety-related behavior [45,46]. The second experiment tested the effects of a CB1 agonist (CP55940) and antagonist (rimonabant), alongside alcohol and diazepam as benchmark anxiolytic agents in these lines [40], on the expression of FPS in HAP1 and LAP1 male and female mice. It was hypothesized that HAP1 mice would be more sensitive to the anxiolytic and anxiogenic effect of CP55940 and rimonabant, respectively. This hypothesis was based on our own findings of differential sensitivity to LY2183240 in HAP2 vs. LAP2 mice [43] and on the findings of Cippitelli et al. [47], who showed that selectively bred Marchigian Sardinian alcohol-preferring rats were more sensitive to the effects of a CB1 receptor antagonist on alcohol self-administration and conditioned reinstatement and had greater CB1 receptor mRNA expression in several brain regions. Finally, we also monitored the estrous cycle of females throughout the experiments to determine whether fluctuations in the estrous cycle influenced the expression of FPS. 

## 2. Materials and Methods

### 2.1. Subjects

Subjects were alcohol-naïve male and female HAP1 and LAP1 selectively bred mice bred from a progenitor population of outbred HS/Ibg mice (Institute of Behavioral Genetics, Boulder, CO) at the Indiana Alcohol Research Center in Indianapolis, IN [48]. Subjects were derived from 43 different HAP1 families from the 42^nd^, 45^th^, and 47^th^ generation of selection and 40 different LAP1 families from the 27^th^ generation of selection. Multiple replications of the experiment were conducted. In each of these replications, subject representation was balanced across line and sex to the best extent possible. On the first day of each experiment, mice were between 54–102 (experiment 1) and 55–165 (experiment 2) days old. Mice were housed in polycarbonate cages (29.2 × 19.0 × 12.7 cm) with aspen wood shavings in groups of 2–4 per cage. Ambient room temperature was maintained at 21 ± 2°C. Mice had free access to food and water in the home cage at all times, except when testing procedures took place. Experimental procedures were conducted during the light phase of a 12:12 light:dark cycle (lights off at 19:00).

All experimental procedures were approved by the Purdue Animal Care and Use Committee (approved protocol #1112000327) and were conducted in accordance with the Guide for the Care and Use of Laboratory Animals. 

### 2.2. Drugs

CP55940 (Sigma-Aldrich, St Louis, MO, USA) was dissolved in 10% DMSO (Malinckrodt, Hazelwood, MO, USA) and administered in doses of 0.01 and 0.03 mg/kg (10.0 ml/kg). Rimonabant (Cayman Chemical, Ann Arbor, MI, USA) was dissolved in 5% Tween 80 (Sigma-Aldrich) and administered in doses of 1.5 and 3.0 mg/kg (10.0 ml/kg). Diazepam (Sigma-Aldrich) was dissolved in a 10% or 45% 2-Hydroxypropyl-Beta-cyclodextrin solution (Sigma-Aldrich) and administered in doses of 4.0 and 6.0 mg/kg (10.0 ml/kg). These doses were chosen because we previously reported selective anxiolytic effects of 4.0 mg/kg diazepam on the expression % FPS in HAP1/2 and LAP1/2 male and female mice [40]. Thus, we chose the 4.0 mg/kg and a higher dose of 6.0 mg/kg diazepam as a benchmark against which to test the effects of the EC drugs. Alcohol was diluted from a 95% (v/v) solution to a concentration of 20% (v/v) with physiological saline (0.9%) and administered at a dose of 1.5 g/kg of body weight in an injection volume of 9.45 ml/kg. This dose of alcohol was used as a benchmark because it reduced the expression of % FPS in HAP1/2 male and female mice, but not in LAP1/2 male and female mice, without producing non-specific motor effects [40]. 

### 2.3. Acoustic Startle Apparatus

FPS was assessed using a Kinder Scientific (Poway, CA, USA) Startle Monitor system composed of 8 dark sound-attenuated chambers, each containing a weight-sensitive platform located approximately 26 cm from a ceiling-mounted speaker and 6 W light bulb. Startle stimuli consisted of broadband noise bursts with a flat frequency distribution from 3–30 kHz (100dB). Subjects were placed individually into open-air Plexiglas holders (4x8.5 x15 cm) with metal rod floors (rod diameter 0.3175 cm each rod separated by 0.465 cm). Scrambled electric foot shock was administered through the metal rod floors of each holder using dual programmable shocker units. The holders were affixed on top of the weight-sensitive platforms during acoustic startle conditioning and test sessions. Subjects’ startle reflex in response to acoustic stimuli was measured as the maximum amount of force in Newtons exerted against the platform during the 200 ms after the onset of each stimulus. Each of the weight-sensitive platforms contain a piezoelectric sensor disc which accounts for and negates body weight when measuring startle reflex response by converting and recording only the acceleration (or increase in force). The startle chambers were completely dark, except when the 6 W light stimulus was presented during FPS conditioning and test sessions. A constant 70 dB background white noise was also presented throughout the sessions via speakers within the chambers. Holders were cleaned with warm water and metal rod floors were cleaned with 70% ethanol between each mouse.

### 2.4. FPS Procedures

During the conditioning session and following a 5 min habituation period, fear-conditioned groups received 20 trials of a 30 sec, 6 W light stimulus paired with a 0.5 sec, 0.8 mA foot shock (2 min intertrial interval). The foot shock occurred during the last 0.5 sec of the light stimulus presentation. Control groups (Experiment 1 only) received the same number and sequence of light and shock presentations as the fear-conditioned group but these stimuli were explicitly unpaired during each of the 20 two minute intervals (randomized interstimulus range: 30–90 sec). The FPS test session consisted of a 5 min habituation period followed by 36 total trials (2 min ITI) presented on a random schedule (range: 10–120 sec) to reduce habituation to any single trial type. Twelve of the trials were blank (no stimuli; 40 msec), 12 were noise-alone (100 dB, 40 msec), and 12 were light (6 W, 30 sec) + noise (100 dB, 40 msec). On light + noise trials, the noise stimulus was presented immediately after the light stimulus ended. The FPS conditioning and testing parameters were based on our previous work in HAP1/LAP1 mice [39].

### 2.5. Brain Tissue Preparation and Analysis

Brains were rapidly extracted and hand-dissected to obtain bi-lateral tissue from the PFC, AMG, and HIP using defined brain delineations based on the mouse brain atlas of Paxinos and Franklin [49]. In pilot studies using this technique, average brain weight for the PFC, AMG, and HIP (both dorsal and ventral) were 0.062±0.005 g, 0.051±0.004 g, and 0.072±0.005 g, respectively. Tissues were immediately placed into 1.5 mL microcentrifuge tubes containing an ice cold 0.5% fatty acid-free bovine serum albumin (BSA) (Sigma-Aldrich, St Louis, MO, USA) and 1x Krebs-Ringer-HEPES (KRH) buffer (120 mM NaCl, 4.7 mM KCl, 2.2 mM CaCl_2_, 10 mM HEPES, 1.2 mM KH_2_PO_4_, 1.2 mM MgSO_4_, pH 7.4) solution. Tissues were then washed twice with additional ice cold 0.5% fatty acid-free BSA KRH solution and once with ice cold plain KRH before being placed into an ice cold 1:1 methanol:acetonitrile (Sigma-Aldrich) solution and homogenized using disposable pellet pestles. Arachidonoyl ethanolamide-d8 (0.001 mM; Cayman Chemical) was then added to the homogenized tissue before centrifugation at 13,300 × g and 4 °C. Following centrifugation, supernatant was pipetted out and dried with argon before being stored at −80 °C until LC-MS analysis could be performed. We have previously reported on the optimization and validation of this quantitative method for detecting AEA and 2-AG in mouse brain [50]. 

### 2.6. Estrous Cycle Monitoring

Daily samples of vaginal cells were obtained from each female mouse, beginning 10 days prior to the start of the conditioning session and ending with a final sample collected immediately after the final FPS testing session. The mice were gently and briefly lifted by the tail and a flexible cotton-tipped nasopharyngeal swab (VWR International, West Chester, PA) moistened with deionized water was used to collect cells from the exterior of the vagina. The cells were then smeared onto a standard glass slide, allowed to air dry, and then examined via light microscopy. The approximate stage of estrous (proestrus, estrus, metestrus, or diestrus) was determined by the type and proportions of cells present [51]. Male mice were manipulated in a fashion similar to females and underwent “sham swabbing” to control for handling effects on behavior and neurochemistry.

### 2.7. Study Procedures

#### 2.7.1. Experiment 1: FPS and EC Brain Levels in Male and Female HAP1 and LAP1 Mice

One-hundred and forty-five HAP1 (73 males, 72 females) and 109 LAP1 (52 males, 57 females) mice were randomly assigned to either a fear-conditioned (FC; paired light + shock), control (CON; unpaired light and shock), or no-shock group (NS; light only). Estrous swabbing was performed at 12 noon on each of the 10 days prior to the conditioning session. On the 11^th^ day of swabbing, mice were conditioned and then swabbed immediately following the conditioning session. Twenty-four hours after the start of the conditioning session, all mice were tested for FPS. Immediately following the testing session, mice were weighed, swabbed, and rapidly sacrificed via cervical dislocation. Brains were then immediately removed, dissected, processed, and stored at −80 °C until LC-MS analysis could be completed.

#### 2.7.2. Experiment 2: Effects of CP55940, rimonabant, alcohol, and diazepam on the expression of FPS in male and female HAP1 and LAP1 mice

Two-hundred and forty-nine HAP1 (114 males, 135 females) and 177 LAP1 (102 males, 75 females) mice were randomly assigned to one of the eight following drug pretreatment groups: Saline, CP55940 (0.01 or 0.03 mg/kg), rimonabant (1.5 or 3.0 mg/kg), diazepam (4.0 or 6.0 mg/kg), or 1.5 g/kg alcohol. Estrous swabbing was performed at 12 noon on each of the 10 days to prior to the conditioning session and on the 6 days between testing sessions 2 and 3. On the 11^th^ day of swabbing, mice were conditioned and then swabbed immediately following the conditioning session. All mice were tested for FPS 24 (Test 1), 48 (Test 2), or 216 h (9 days) after the start of the fear-conditioning session. Thirty minutes prior to FPS Test 1 and 2, mice were pretreated with their assigned drug. The purpose of the third FPS test was to assess the effects of prior drug treatments on the persistence of the FPS response in a drug-free state. Mice were swabbed immediately following all FPS test sessions.

### 2.8. Statistical Analyses

All 12 startle responses on each trial type (noise-alone, light + noise) were averaged for each mouse. The % FPS measure was obtained using proportional change scores calculated using the following formula: (((startle amplitude on light + noise trials – startle amplitude on noise-alone trials)/startle amplitude on noise-alone trials) × 10). Thus, % FPS is a sensitive measure that adjusts for individual and group differences (e.g., possible non-specific effects of drug treatment) in startle response magnitude that may be observed on noise-alone and light + noise trials [52].

Mice were removed from all analyses if their average startle response on both the noise-alone and light + noise trials did not exceed the average value obtained on blank (no stimulus) trials on any FPS test (for experiment 2, subjects were removed from all analyses if they failed the criterion on at least 1 of the 3 FPS tests). Applying this criterion resulted in the removal of 16 subjects from analyses in experiment 1 and 90 subjects in experiment 2. For experiment 2, the number of subjects that met the criterion on test 1, 2, or 3 only, on both tests 1 and 2, on both tests 1 and 3, on both tests 2 and 3, or on all three tests in each drug treatment group is shown as follows: Saline, test 1 (*n* = 1), test 2 (*n* = 2), test 3 (*n* = 2), test 1 and 2 (*n* = 8), test 1 and 3 (*n* = 1), test 2 and 3 (*n* = 3), all three tests (*n* = 11); CP 0.01 mg/kg, test 2 (*n* = 1), test 1 and 2 (*n* = 2), test 1 and 3 (*n* = 2), test 2 and 3 (*n* = 3), all three tests (*n* = 5); CP 0.03 mg/kg, test 1 (*n* = 2), test 3 (*n* = 2), test 1 and 2 (*n* = 1), test 1 and 3 (*n* = 1), test 2 and 3 (*n* = 2), all three tests (*n* = 6); rimonabant 1.5 mg/kg, test 1 (*n* = 1), test 2 (*n* = 1), test 1 and 2 (*n* = 1), test 2 and 3 (*n* = 1), all three tests (*n* = 2); rimonabant 3.0 mg/kg, test 1 (*n* = 1), test 2 (*n* = 1), test 3 (*n* = 1), test 1 and 2 (*n* = 1), test 2 and 3 (*n* = 1); alcohol 1.5 g/kg, test 2 (*n* = 3), test 1 and 3 (*n* = 1), all three tests (*n* = 4); diazepam 4.0 mg/kg, test 1 (*n* = 2), test 2 (*n* = 1), test 3 (*n* = 2), test 1 and 2 (*n* = 1), all three tests (*n* = 5); diazepam, 6.0 mg/kg, test 1 (*n* = 2), test 1 and 3 (*n* = 2), test 2 and 3 (*n* = 1). A Chi-Square test conducted on the distribution of subjects removed, based on which tests they met criterion for removal, did not differ in any treatment group. These data indicate that removal of mice was not disproportionate across the three FPS tests, the first two of which mice received drug treatments. 

Mean analyte (AEA and 2-AG) concentration (pmole/μL) was determined for each brain region (PFC, AMG, and HIP) of each subgroup (line, sex, conditioning group) of mice. Individual analyte concentration values for each subject’s brain regions were compared to the subgroup mean of the corresponding brain region. If the absolute difference between a subject’s analyte concentration value and the overall subgroup mean for the corresponding brain region was greater than 2 standard deviations from that subgroup mean, then that subject’s analyte concentration value for that specific brain region was subjected to the Dixon Extreme Score Test [53]. If the value passed as an outlier, the subject was removed from all analyses. Outlier analyses resulted in the removal of 11 subjects from analyses in experiment 1.

Data were analyzed using analysis of variance (ANOVA) with line (HAP, LAP), sex (male, female), conditioning group (FC, CON, NS), and estrous stage ((proestrus (P), estrus (E), metestrus (M), or diestrus (D)), where applicable. In some cases only highest order interactions are reported from the overall ANOVAs to simplify data presentation. Post-hoc analyses included lower-order ANOVAs and Tukey’s tests, as appropriate. Pearson product moment correlation coefficients were generated to assess relationships between variables. Probability values ≤ 0.05 were considered to be significant. 

## 3. Results

### 3.1. Experiment 1: FPS and AEA and 2-AG Brain Levels in Male and Female HAP1 and LAP1 Mice

#### 3.1.1. % FPS

**Figure 1:**
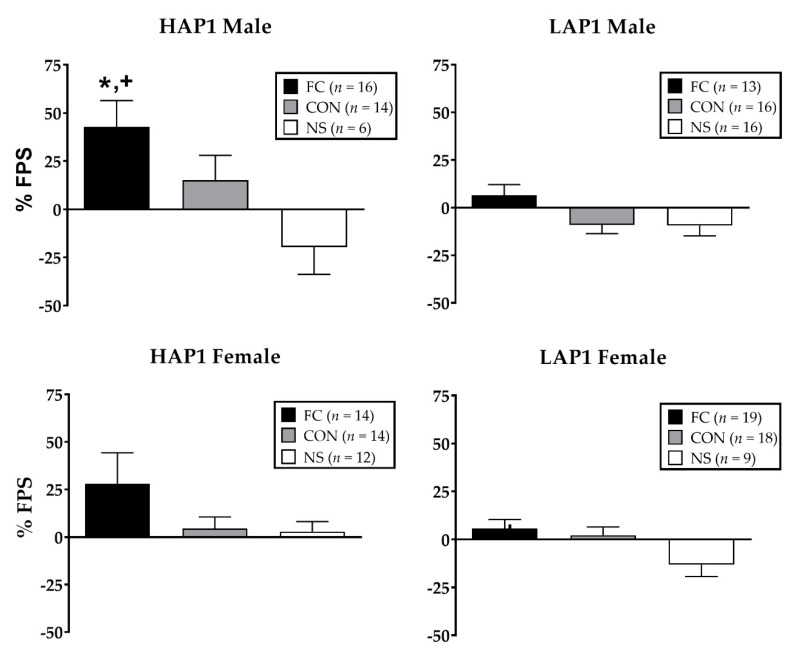
Mean (± SEM (standard error of the mean)) % FPS (fear-potentiated startle) in HAP1 (high alcohol preferring; left panels) and LAP1 (low alcohol preferring; right panels) mice in the fear-conditioned (FC; paired light +shock), control (CON; unpaired light and shock), and no-shock (NS; light only) groups for experiment 1. *p < 0.01, HAP1 > LAP1; + *p* < 0.01, FC > CON, NS (no-shock group).Analysis of % FPS in fear-conditioned HAP1 and LAP1 females as a function of their estrous stage on the FPS test day indicated a main effect in LAP1 mice only (F(3,15) = 4.7, *p* < 0.05). Tukey’s post-hoc analyses indicated that % FPS of LAP1 mice in D stage (36.6 ± 9.2, *n* = 3) was significantly greater than those in E (−3.6 ± 5.6, *n* = 8, *p* = 0.01) and M (2.8 ± 7.1, *n* = 5, *p* < 0.05) stages.

ANOVA (Line × Sex × Conditioning Group) indicated significant main effects of line (F (1,155) = 7.7, *p* < 0.01; HAP1 > LAP1) and conditioning group (F (2,155) = 10.2, *p* < 0.001; FC > CON, NS). Data in Figure 1 are shown separated by sex despite the lack of significant sex differences in FPS. 

#### 3.1.2. AEA and 2-AG in PFC, AMG, and HIP Following FPS Testing

##### AEA

**Figure 2:**
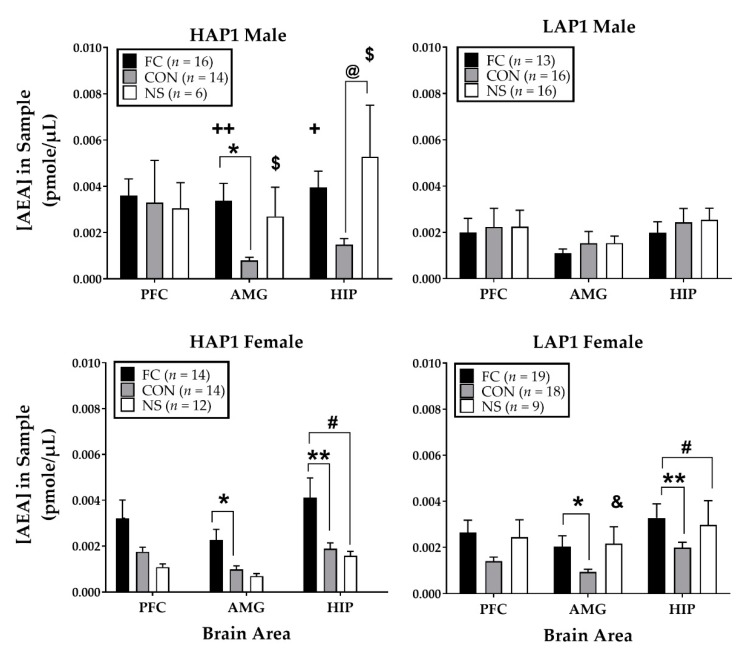
Mean (±SEM) concentration of AEA (the endocannabinoids, anandamide) in the prefrontal cortex (PFC), amygdala (AMG), and hippocampus (HIP) immediately after the FPS test in HAP1 (left panels) and LAP1 (right panels) male and female mice in the fear-conditioned (FC; paired light + shock), control (CON; unpaired light and shock) and no-shock (NS; light only) groups for experiment 1. +*p* = 0.05, ++*p* = 0.01 FC males HAP1>LAP1; *p < 0.05, ** *p* < 0.01 FC > CON (collapsed across line in females only); #*p* < 0.05, females FC > NS (collapsed across line); male HAP1 @*p* < 0.05, NS > CON; &*p* < 0.05, NS females LAP1 > HAP1; $*p* < 0.05, NS HAP1 males > females.

For AEA following FPS testing (Figure 2), ANOVAs (Line × Sex × Conditioning Group) for each brain area indicated significant three-way interactions of Line × Sex × Conditioning Group in the AMG (F (2,155) = 3.9, *p* < 0.05) and HIP (F (2,155) = 3.4, *p* < 0.05). 

Follow-up analyses of the three-way interaction in the AMG (Line × Sex ANOVAs within each Conditioning Group) yielded Line effects in the AMG (F (1,58) = 5.4, *p* < 0.05; HAP1 > LAP1) and HIP (F (1,58) = 4.2, *p* < 0.05; HAP1 > LAP1) of FC groups. Line × Sex interactions were also found in the AMG (F (1,39) = 5.7, *p* < 0.05] and HIP [F (1,39) = 5.4, *p* < 0.05) of the NS groups. Follow-up of these interactions (one-way ANOVAs of Sex within each Line) in NS groups yielded main effects of Sex in HAP1 NS groups, as follows: AMG (F (1,16) = 5.2, *p* < 0.05; males > females) and HIP (F (1,16) = 5.7, *p* < 0.05; males > females). ANOVAs of Line within each sex in NS groups indicated a main effect of Line in the AMG of NS females only (F (1,19) = 5.1, *p* < 0.05; LAP1 > HAP1). 

Additional follow up ANOVAs (Line × Conditioning Group within each sex) for the AMG and HIP were conducted to explore the overall three-way interactions. 

For the AMG, in females, there was a main effect of Conditioning Group (F (2,80) = 5.6, *p* < 0.01) and Tukey’s post-hoc indicated AEA levels in the AMG were significantly higher in the FC females compared to the CON females (*p* < 0.05; collapsed across Line). In males, there was a Line × Conditioning Group interaction (F (2,75) = 4.5, *p* = 0.01). Follow-up to this interaction in males (one-way ANOVAs of Line within each Conditioning Group) indicated significantly greater AEA in the AMG of HAP1 than LAP1 males in the FC groups (F (1,27) = 7.0, *p* = 0.01). Follow up ANOVAs of Conditioning Group within each Line indicated a main effect of Conditioning Group in HAP1 male mice (F (2,33) = 4.5, *p* < 0.05) and Tukey’s post-hoc analyses indicated AEA levels were significantly higher in the FC compared to the CON (*p* < 0.05) males.

For the HIP, in females, there was a main effect of Conditioning Group (F (2,80) = 5.8, *p* < 0.01) and Tukey’s post-hoc indicated AEA levels were significantly higher in the FC females compared to the CON females (*p* < 0.01; collapsed across Line) and higher compared to the NS females (*p* < 0.05; collapsed across Line). In males, there was a Line × Conditioning Group interaction (F (2,75) = 3.9, *p* < 0.05). Follow-up to this interaction in males (one-way ANOVAs of Line within each Conditioning Group) indicated significantly greater AEA in the HIP of HAP1 than LAP1 males in the FC groups (F (1,27) = 4.9, *p* < 0.05). Follow up ANOVAs of Conditioning Group within each Line indicated significant main effects of Conditioning Group in HAP1 males (F (2,33) = 4.5, *p* < 0.05) only. Tukey’s post-hoc analyses indicated that AEA levels in HAP1 males were significantly higher in the NS than the CON group (*p* < 0.05). 

Correlations between % FPS within each treatment group and AEA levels in each brain area yielded a significant positive correlation between AEA and % FPS in the PFC in the CON group (r^2^ = 0.31, *p* < 0.05, *n* = 62). Further breakdown of this relationship as a function of Line indicated a positive correlation in the HAP1 CON group only (r^2^ = 0.46, *p* < 0.05, *n* = 28). 

Analyses of AEA levels (pmol/µL) in the PFC, AMG, and HIP in HAP1 and LAP female mice within each conditioning group as a function of estrous stage yielded a significant main effect in the HIP of FC LAP1 mice (F(3,15) = 3.7, *p* < 0.05). Tukey’s post hoc indicated that AEA was significantly greater in LAP1 mice in M stage (0.006 ± 0.001, *n* = 5) compared to P stage (0.001 ± 0.0003, *n* = 3, *p* < 0.05).

##### 2-AG

**Figure 3:**
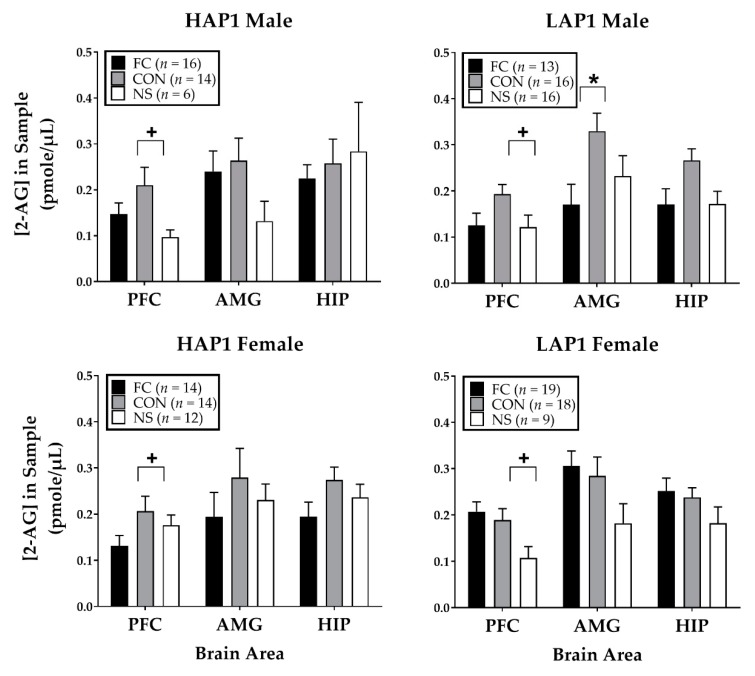
Mean (±SEM) 2-AG expression immediately following FPS testing in HAP1 (left panels) and LAP1 (right panels) male and female mice in the fear-conditioned (FC; paired light + shock), control (CON; unpaired light and shock) and no-shock (NS; light only) groups for experiment 1. +*p* < 0.01, CON>NS (collapsed across line and sex); *p < 0.05, male LAP1 CON > FC.

For 2-AG following FPS testing (Figure 3), ANOVAs (Line × Sex × Conditioning Group) for each brain area indicated a significant main effect of Conditioning Group (F (2,155) =7.2, *p* = 0.001) in the PFC. Tukey’s post-hoc indicated 2-AG was significantly higher in the CON compared to the NS groups (*p* < 0.01). The AMG ANOVA indicated a significant Line × Sex × Treatment group interaction (F (2,155) = 3.3, *p* < 0.05). Follow-up ANOVAs of Line × Conditioning group within each sex indicated no significant effects. Line × Sex ANOVAs within each Conditioning Group yielded a significant Line × Sex interaction in the FC group (F (1,58) = 4.4, *p* < 0.05). Follow-up one-way ANOVAs of Line within each Sex indicate the interaction was due to a Line effect close to significance in the AMG of females (F (1,31) = 3.7, *p* = 0.06) but not in males. Conditioning Group × Sex ANOVAs within each Line indicated a two-way interaction in LAP1 mice (F (2,85) = 3.4, *p* < 0.05). Follow-up one-way ANOVAs of Conditioning Group within each Sex yielded a main effect in male LAP1 mice (F (2,42) = 3.5, *p* < 0.05). Tukey’s post-hoc showed that the male LAP1 CON group had significantly higher 2-AG in the AMG compared to the male LAP1 FC group (*p* < 0.05). 

Correlations between % FPS within each treatment group and 2-AG levels in each brain area yielded no significant correlations.

Analyses of 2-AG levels (pmol/µL) in the PFC, AMG, and HIP in HAP1 and LAP1 female mice within each conditioning group as a function of estrous stage yielded a significant main effect in the PFC of NS LAP1 mice (F (3,5) = 5.8, *p* < 0.05). Tukey’s post hoc was invalid due to only 1 subject in E stage. However, the main effect was due to greater 2-AG levels in LAP1 mice in D stage (0.18 ± 0.03, *n* = 3) compared to M stage (0.04 ± 0.03, *n* = 2).

### 3.2. Experiment 2: Effects of CP55940, Rimonabant, Alcohol, and Diazepam on the Expression of FPS in Male and Female HAP1 and LAP1 Mice % FPS

**Figure 4:**
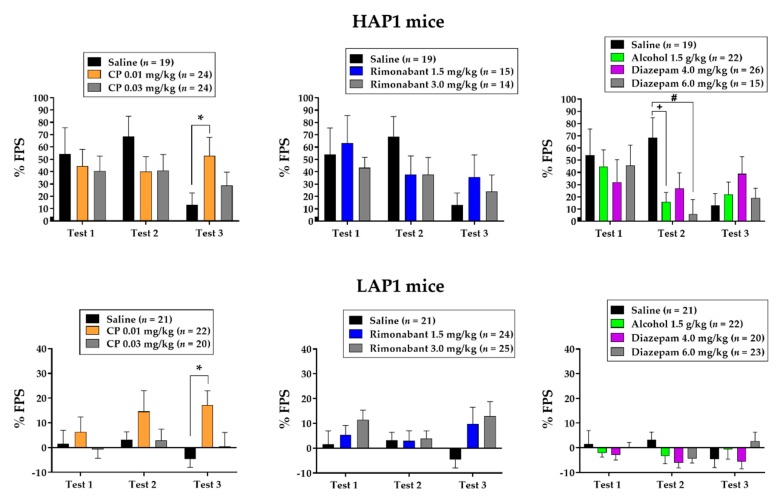
Mean (±SEM) % FPS in HAP1 male and female (top panels) and LAP1 male and female (bottom panels) mice that received saline, 0.01 mg/kg and 0.03 mg/kg CP55940 (left panels), 1.5 mg/kg and 3.0 mg/kg rimonabant (middle panels), or 1.5 g/kg alcohol, 4.0 mg/kg diazepam, and 6.0 mg/kg diazepam (right panels). Drugs were given 30 min prior to Test 1 and Test 2 which occurred 24 and 48 hrs after fear-conditioning, respectively. Test 3 occurred 216 hrs (9 days) after Test 2 in a drug-free state. *p < 0.01 CP55940 0.01 mg/kg > Saline (collapsed across Line); +*p* < 0.05, Saline > Alcohol 1.5 g/kg; #*p* < 0.01, Saline>Diazepam 6.0 mg/kg.

Figure 4 shows the same data from the saline-treated group in each panel vs. CP55940 (left panels), rimonabant (middle panels), and alcohol and diazepam (right panels) to facilitate comparisons. ANOVAs (Line × Sex × Treatment) were conducted separately for CP55940 vs. saline, rimonabant vs. saline, and alcohol and diazepam vs. saline on each test day.

#### 3.2.1. CP55940 

The ANOVAs indicated main effects of Line (HAP1 > LAP1) on all three tests (Fs (3,118) > 11.3, *p* < 0.01) and a main effect of Treatment on Test 3 (F (2,118) = 4.6, *p* = 0.01). Tukey’s post-hoc indicated that FPS was significantly greater in the 0.01 mg/kg CP55940-treated groups compared to saline-treated groups (*p* < 0.01). 

#### 3.2.2. Rimonabant

The ANOVAs indicated main effects of Line (HAP1 > LAP1) on all three tests (Fs (1,106) > 8.3, *p* < 0.01) and a Line × Sex × Treatment interaction on test 3 (F (2,106) = 3.2, *p* < 0.05). Follow-up ANOVAs of Line × Treatment within each sex indicated a Line × Treatment interaction in males (F (2,57) = 3.1, *p* = 0.05). One-way ANOVAs of Treatment within each Line showed no significant effects but analyses of Line within each Treatment group indicated the interaction was due to significant line differences (HAP1 > LAP1) in the saline-treated (F (1,19) = 7.6, *p* < 0.05) and rimonabant 1.5 mg/kg-treated (F (1,19) = 6.8, *p* < 0.05) group, but not in the rimonabant 3.0 mg/kg-treated group. 

#### 3.2.3. Alcohol and Diazepam

The ANOVAs indicated main effects of Line (HAP1 >LAP1) on all three tests (Fs (1,152) > 18.1, *p* < 0.001) and Line × Treatment interaction on test 2 (F (3,152) = 3.2, *p* < 0.05). One-way ANOVAs of Treatment within each Line yielded a main effect of Treatment in HAP1 (F (3,78) = 4.2, *p* < 0.01) but not LAP1 mice. Tukey’s post-hoc indicated that FPS was significantly lower in the alcohol 1.5 g/kg-treated (*p* < 0.05) and diazepam 6.0 mg/kg-treated (*p* = 0.01) groups compared to saline-treated groups. Analyses of Line within each Treatment showed Line effects in saline-treated (F (1,38) = 16.9, *p* < 0.001), alcohol 1.5 g/kg-treated (F (1,42) = 5.1, *p* < 0.05), and diazepam 4.0 mg/kg-treated (F (1,44) = 5.0, *p* < 0.05) groups but not in the diazepam 6.0 mg/kg-treated group. 

Analysis of % FPS in HAP1 and LAP1 females as a function of estrous stage on Test 1 indicated a main effect close to significance in LAP1 mice (F (3,71) = 2.7, *p* = 0.055). Tukey’s post-hoc indicated that % FPS of LAP1 mice in D stage (8.7 ± 3.8, *n* = 15) was significantly greater than those in P stage (−3.9 ± 2.8, *n* = 27, *p* < 0.05). Analysis of % FPS in HAP1 females as a function of estrous stage on Test 2 indicated no significant effect. The same analysis on Test 3 yielded a main effect of estrous stage in LAP1 mice (F (3,71) = 7.7, *p* < 0.001). Tukey’s post-hoc indicated that % FPS of LAP1 mice in D stage (30.1 ± 8.1, *n* = 8) was significantly greater than mice in M (−19.5 ± 6.4, *n* = 13, *p* < 0.001), P (1.4 ± 3.9, *n* = 35, *p* = 0.01), and E (2.5 ± 5.3, *n* = 19, *p* < 0.05) stages. In addition, % FPS of LAP1 mice in E and P stages was greater than those in M (*p* < 0.05) stage.

## 4. Discussion

The results of Experiment 1 suggest that ECS function in response to conditioned fear stress (FPS) is associated with genetic propensity toward alcohol preference in HAP1 and LAP1 male and female mice. FPS was greater in male and female HAP1 than LAP1 mice, as previously reported [39,40,41]. Although there were no sex differences in FPS expression, in fear-conditioned mice, AEA levels in the AMG and HIP of males were greater in HAP1 than LAP1 mice. No line difference in AEA was seen in female fear-conditioned mice. The results of Experiment 2 did not support the hypothesis that HAP1 mice would be more sensitive to CB1 receptor ligands, based on work in Marchigian Sardinian alcohol-preferring rat lines [47]. 

There were differences in the pattern of AEA levels as a function of line, sex, conditioning group, and brain area. However, the principal finding from Experiment 1 is that HAP1 mice in fear-conditioned groups showed greater AEA levels in the AMG and HIP than LAP1 mice, suggesting that AEA response to fear-conditioning stress may be genetically correlated with line differences in FPS expression. Similar line differences were not seen in the control or no shock groups, suggesting that the line differences in AEA in these brain regions are a specific consequence of fear-conditioning. The AMG is a critical brain region that regulates the acquisition and expression of FPS [35] and studies have shown that AEA is reduced following stress in amygdalar brain regions [22,29] due to a rapid induction of FAAH [54,55]. In general, higher levels of AEA in HAP1 than LAP1 mice appears contradictory in relation to findings in the literature where pharmacological enhancement of AEA levels via EC uptake inhibitors or FAAH genetic deletion was associated with anxiolytic effects in rodent models [28,29,31]. These line differences may be due to differential expression of FAAH or CB1 [32] in the AMG and HIP between HAP1 and LAP1 mice. This suggestion is supported by our prior findings that LY2183240, an EC uptake inhibitor, reduced the expression of FPS in HAP1/2 but not LAP1 mice [43].

There were a few other general patterns with respect to group differences in AEA that were line- and sex-specific. In the AMG, females from both lines and HAP1 males showed higher AEA in the FC groups compared to CON groups. A similar finding was found in the HIP of females from both lines, where AEA was higher in the FC groups compared to CON and NS groups. In general, the direction of these effects as a function of conditioning group (FC > CON) may seem contradictory in light of studies showing that AEA is reduced in the brain following stress [28,29]. The FPS test is more anxiogenic for FC groups than CON groups, as measured by the anticipatory fear response (FPS), and therefore may elicit greater recruitment of stress-responsive mechanisms. In our prior work we found evidence for this possibility in LAP2 male mice, as follows: FC mice showed higher levels of corticosterone (CORT) compared to CON mice immediately following the FPS test [41]. Here, the group difference (FC > CON) in AEA levels in HAP1 males only is interesting in light of our prior work in these lines, where we found stronger FPS in HAP1/2 males than HAP1/2 females [40,41], and data in the literature showing that male animals are more susceptible to stress-related anxiety in animal models of PTSD [56,57]. 

It’s also interesting to note a similar u-shaped pattern of AEA in the AMG and HIP of HAP1 males and LAP1 females, such that the level of AEA is lower in CON groups and similar between FC and NS groups (Figure 2). In fact, AEA in the HIP of NS HAP1 males was significantly greater than AEA in the CON group (Figure 2, upper left panel, significance indicated by @ symbol). This pattern might be due to a non-specific stress-related response to acoustic startle stimuli in the NS groups during the FPS test. There were also line- and sex- dependent differences in AEA found in NS groups worth noting. AEA in the AMG and HIP was higher in HAP1 males than females and higher in LAP1 females compared to HAP1 females. These observed differences may be related to line- or sex-dependent ECS function [58,59] or estradiol status within females, as estradiol has been shown to be particularly important in regulating FAAH [60]. 

With respect to 2-AG, levels in the PFC were higher overall in most CON groups compared to the NS groups. This result is interesting in the context of findings showing that stress-induced elevation of 2-AG is associated with greater resistance to traumatic stress [23], as 2-AG plays a role in biochemical homeostatic recovery from stress insults [61]. Thus, elevated 2-AG in the PFC may indicate a stress-related adaptive response to cognitive processing of a potential threat in response to contextual stimuli previously associated with footshock in the chamber and/or unpredictable footshock. In addition, in the AMG, we found line- and sex-specific effects on 2-AG levels. Male LAP1 (but not HAP1) mice showed higher 2-AG levels in the CON group compared to the FC group. This result may reflect greater stress-adaptive ECS functions in the AMG of LAP1 males and may possibly relate to their resistance to fear-conditioning (compared to HAP1 males), as measured by FPS. Similar to our prior work [40,41], Figure 1 shows a trend toward greater FPS in HAP1 males compared to females, but this was not found to be statistically significant. 

As mentioned in the introduction, stress produces different effects on AEA and 2-AG levels in the brain, which likely reflects time-dependent changes in the induction of catabolic enzymes that regulate neuronal excitability and recovery from stress [32]. We assessed AEA and 2-AG in brain regions important for processing emotional memory [62,63] following a test of conditioned fear, which elicits a stress response, as measured by activation of the HPA axis [41]. We know from our prior work in the HAP/LAP lines that HPA axis response to fear-conditioning stress, as measured by CORT, is a biological marker for genetic vulnerability to develop fear-related behavior [41]. LAP2 mice show higher CORT levels following fear-conditioning and testing than HAP2 mice. These findings are relevant in the context of interpreting the current results because there are bidirectional relationships between the ECS and the HPA axis function [54,64,65], stress-related behaviors [66], and emotional memory [22,67,68]. Under steady-state conditions, the ECS serves as a tonic regulator of HPA axis activity. Glucocorticoids released in response to stress bind to the corticotropin releasing factor type 1 receptor, which mobilizes FAAH, resulting in a reduction of AEA [69], which in turn disinhibits HPA axis activity. At the same time, glucocorticoids trigger the synthesis and release of ECs which bind to CB1 receptors, which in turn dampen HPA axis activity [69,70]. Bitencourt et al. showed that ECs influence on expression of contextual fear memory in rats via CB1 receptors depends on glucocorticoid activity [71]. Thus, we hypothesize that the line differences observed in AEA and 2-AG in the present study are likely related to line differences in HPA axis function between the lines. Future work is warranted to explore ECS-HPA axis interactions in these lines to identify selective pharmacological interventions to reduce both alcohol drinking and fear-related behaviors. 

In Experiment 2, drugs were administered prior to two consecutive FPS tests based on our prior finding with the EC uptake inhibitor LY2183240 [43]. In that study, LY2183240 reduced FPS only after a second administration prior to a second FPS test in HAP1/2 but not LAP1 mice, possibly indicating that the fear extinction process requires repeated activation of the ECS. In the current study, we administered a third, drug-free, FPS test 7 days after the second FPS test (9 days after conditioning) to assess the persistence of the FPS response and whether the previous drug treatments administered prior to FPS testing altered the expression of FPS in a drug-free state. The results of Experiment 2 did not support the hypothesis that HAP1 mice would be more sensitive to the pharmacological effects of CP59940 and rimonabant compared to LAP1 mice. This outcome could be due to genetic differences between the experimental subjects tested here (HAP1 and LAP1) compared to those in our previous study (both HAP1/2 and LAP1) with LY2183240 [43]. In addition, it should be noted that the overall magnitude of % FPS in LAP1 mice was relatively low on test 1, which could have reduced the range in which to observe pharmacological effects of the drugs on the expression of FPS. However, there was an interesting effect seen on the third, drug-free test, where FPS was significantly greater in the 0.01 mg/kg CP55940-treated groups (collapsed across line and sex) compared to saline-treated groups (Figure 4, left panels). This result suggests that increasing activation of CB1 receptors during the FPS test may be anxiogenic and/or interfere with learning-mechanisms (e.g., extinction) that maintain the expression of FPS. This idea is consistent with previous findings where higher or repeated doses resulted in increased anxiety in both mice [72,73] and rats [74]. Future work is necessary to specifically explore effects of ECS drugs on stress-related behaviors and extinction learning in these lines.

In the current study, we included alcohol (1.5 g/kg) and two doses of diazepam (4.0 mg/kg, 6.0 mg/kg) as reference drugs known to produce anxiolytic effects in the FPS procedure [40]. Here, we replicated our prior findings by showing that alcohol reduced the expression of FPS in HAP1 but not in LAP1 mice. We also expand upon our prior findings with diazepam by showing here that HAP1 mice are more sensitive to the anxiolytic effects of 6.0 mg/kg diazepam compared to LAP1 mice. Thus, it appears that the line difference in sensitivity to diazepam’s anxiolytic effects are dose-dependent, as no line difference in response to 4.0 mg/kg was seen here or in our previous report [40]. 

In both experiments, LAP1 females showed significantly greater FPS when tested in the D stage, when estradiol is lower, compared to those tested in the M and E stages, when estradiol is higher [75]. Although the literature contains many conflicting reports on estradiol’s effects on fear- and anxiety-related behavior, our finding in LAP1 females is consistent with at least one report indicating anxiolytic effects of estrogen replacement on contextual fear behavior in rats [76]. Other studies in cycling rats have not found effects of estrous cycle on the expression of FPS [77,78,79]. These findings highlight the importance of monitoring the estrous cycle and, if possible, measuring estradiol when testing both males and females in fear-conditioning studies. 

In summary, we found that mice selectively bred for high alcohol preference, which are vulnerable to develop conditioned fear-related behavior, show differences in AEA and 2-AG levels after expression of conditioned fear-related behavior (FPS) compared to mice selectively bred for low alcohol preference. The findings suggest that ECS brain mechanisms modulate both genetic susceptibility to fear-related behavior and alcohol drinking behavior in these mouse lines. One caveat to this conclusion is that ECS function, and response to CP55940 and rimonabant, was assessed in replicate line 1 mice only. Future studies in the HAP/LAP replicate lines 2 and 3 are warranted to explore how ECS function, as well as ECS-neuroendocrine interactions, influences susceptibility toward fear-related and alcohol-drinking behaviors. These mouse lines represent a relevant pre-clinical animal model to identify selective pharmacotherapeutic treatments for individuals with co-morbid disorders, particularly in individuals with genetic risk factors for AUDs and/or PTSD. 

## References

[1] Allen J.P., Crawford E.F., Kudler H. (2016). Nature and treatment of comorbid alcohol problems and post traumatic stress disorder among American military personnel and veterans. Alcohol Res..

[2] Grant B.F., Saha T.D., Ruan W.J., Goldstein R.B., Chou S.P., Jung J., Zhang H., Smith S.M., Pickering R.P., Huang B. (2016). Epidemiology of DSM-5 Drug Use Disorder: Results From the National Epidemiologic Survey on Alcohol and Related Conditions-III. JAMA Psychiatry.

[3] Blanco C., Xu Y., Brady K., Perez-Fuentes G., Okuda M., Wang S. (2013). Comorbidity of posttraumatic stress disorder with alcohol dependence among US adults: Results from National Epidemiological Survey on Alcohol and Related Conditions. Drug Alcohol Depend..

[4] Sartor C.E., McCutcheon V.V., Pommer N.E., Nelson E.C., Grant J.D., Duncan A.E., Waldron M., Bucholz K.K., Madden P.A., Heath A.C. (2011). Common genetic and environmental contributions to post-traumatic stress disorder and alcohol dependence in young women. Psychol. Med..

[5] Enoch M.A. (2012). The influence of gene-environment interactions on the development of alcoholism and drug dependence. Curr. Psychiatry Rep..

[6] Greenwald M.K. (2018). Anti-stress neuropharmacological mechanisms and targets for addiction treatment: A translational framework. Neurobiol. Stress.

[7] Ney L.J., Matthews A., Bruno R., Felmingham K.L. (2019). Cannabinoid interventions for PTSD: Where to next?. Prog. Neuropsychopharmacol. Biol. Psychiatry.

[8] Spagnolo P.A., Ramchandani V.A., Schwandt M.L., Kwako L.E., George D.T., Mayo L.M., Hillard C.J., Heilig M. (2016). FAAH gene variation moderates stress response and symptom severity in patients with posttraumatic stress disorder and comorbid alcohol dependence. Alcohol Clin. Exp. Res..

[9] Zhou Y., Kreek M.J. (2018). Involvement of activated brain stress responsive systems in excessive and “relapse” alcohol drinking in rodent models: Implications for therapeutics. J. Pharmacol. Exp. Ther..

[10] Devane W.A., Dysarz F.A., Johnson M.R., Melvin L.S., Howlett A.C. (1988). Determination and characterization of a cannabinoid receptor in rat brain. Mol. Pharmacol..

[11] Matsuda L.A., Lolait S.J., Brownstein M.J., Young A.C., Bonner T.I. (1990). Structure of a cannabinoid receptor and functional expression of the cloned cDNA. Nature.

[12] Munro S., Thomas K.L., Abu-Shaar M. (1993). Molecular characterization of a peripheral receptor for cannabinoids. Nature.

[13] Deutsch D.G., Ueda N., Yamamoto S. (2002). The fatty acid amide hydrolase (FAAH). Prostaglandins Leukot Essent Fatty Acids.

[14] Cravatt B.F., Giang D.K., Mayfield S.P., Boger D.L., Lerner R.A., Gilula N.B. (1996). Molecular characterization of an enzyme that degrades neuromodulatory fatty-acid amides. Nature.

[15] Sidhpura N., Parsons L.H. (2011). Endocannabinoid-mediated synaptic plasticity and addiction-related behavior. Neuropharmacology.

[16] Zlebnik N.E., Cheer J.F. (2016). Drug-induced alterations of endocannabinoid-mediated plasticity in brain reward regions. J. Neurosci..

[17] Lu A.T., Ogdie M.N., Jarvelin M.R., Moilanen I.K., Loo S.K., McCracken J.T., McGough J.J., Yang M.H., Peltonen L., Nelson S.F. (2008). Association of the cannabinoid receptor gene (CNR1) with ADHD and post-traumatic stress disorder. Am. J. Med. Genet. B Neuropsychiatr. Genet..

[18] Lopez-Moreno J.A., Echeverry-Alzate V., Buhler K.M. (2012). The genetic basis of the endocannabinoid system and drug addiction in humans. J. Psychopharmacol..

[19] Sloan M.E., Gowin J.L., Yan J., Schwandt M.L., Spagnolo P.A., Sun H., Hodgkinson C.A., Goldman D., Ramchandani V.A. (2018). Severity of alcohol dependence is associated with the fatty acid amide hydrolase Pro129Thr missense variant. Addict. Biol..

[20] Pava M.J., Woodward J.J. (2012). A review of the interactions between alcohol and the endocannabinoid system: Implications for alcohol dependence and future directions for research. Alcohol.

[21] Mizrachi Zer-Aviv T., Segev A., Akirav I. (2016). Cannabinoids and post-traumatic stress disorder: Clinical and preclinical evidence for treatment and prevention. Behav. Pharmacol..

[22] Akirav I. (2013). Cannabinoids and glucocorticoids modulate emotional memory after stress. Neurosci. Biobehav. Rev..

[23] Bluett R.J., Baldi R., Haymer A., Gaulden A.D., Hartley N.D., Parrish W.P., Baechle J., Marcus D.J., Mardam-Bey R., Shonesy B.C. (2017). Endocannabinoid signalling modulates susceptibility to traumatic stress exposure. Nat. Commun..

[24] Hill M.N., Campolongo P., Yehuda R., Patel S. (2018). Integrating endocannabinoid signaling and cannabinoids into the biology and treatment of posttraumatic stress disorder. Neuropsychopharmacology.

[25] Hillard C.J. (2018). Circulating endocannabinoids: From whence do they come and where are they going?. Neuropsychopharmacology.

[26] Morena M., Patel S., Bains J.S., Hill M.N. (2016). Neurobiological interactions between stress and the endocannabinoid system. Neuropsychopharmacology.

[27] Worley N.B., Hill M.N., Christianson J.P. (2018). Prefrontal endocannabinoids, stress controllability and resilience: A hypothesis. Prog. Neuropsychopharmacol. Biol. Psychiatry.

[28] Bluett R.J., Gamble-George J.C., Hermanson D.J., Hartley N.D., Marnett L.J., Patel S. (2014). Central anandamide deficiency predicts stress-induced anxiety: Behavioral reversal through endocannabinoid augmentation. Transl. Psychiatry.

[29] Hill M.N., Kumar S.A., Filipski S.B., Iverson M., Stuhr K.L., Keith J.M., Cravatt B.F., Hillard C.J., Chattarji S., McEwen B.S. (2013). Disruption of fatty acid amide hydrolase activity prevents the effects of chronic stress on anxiety and amygdalar microstructure. Mol. Psychiatry.

[30] Patel S., Kingsley P.J., Mackie K., Marnett L.J., Winder D.G. (2009). Repeated homotypic stress elevates 2-arachidonoylglycerol levels and enhances short-term endocannabinoid signaling at inhibitory synapses in basolateral amygdala. Neuropsychopharmacology.

[31] Moreira F.A., Kaiser N., Monory K., Lutz B. (2008). Reduced anxiety-like behaviour induced by genetic and pharmacological inhibition of the endocannabinoid-degrading enzyme fatty acid amide hydrolase (FAAH) is mediated by CB1 receptors. Neuropharmacology.

[32] Gunduz-Cinar O., Hill M.N., McEwen B.S., Holmes A. (2013). Amygdala FAAH and anandamide: Mediating protection and recovery from stress. Trends Pharmacol. Sci..

[33] Jenniches I., Ternes S., Albayram O., Otte D.M., Bach K., Bindila L., Michel K., Lutz B., Bilkei-Gorzo A., Zimmer A. (2016). Anxiety, stress, and fear response in mice with reduced endocannabinoid levels. Biol. Psychiatry.

[34] Lissek S., van Meurs B. (2015). Learning models of PTSD: Theoretical accounts and psychobiological evidence. Int. J. Psychophysiol..

[35] Davis M., Falls W.A., Campeau S., Kim M. (1993). Fear-potentiated startle: A neural and pharmacological analysis. Behav. Brain Res..

[36] Grillon C. (2008). Models and mechanisms of anxiety: Evidence from startle studies. Psychopharmacology.

[37] Groenink L., Bijlsma E.Y., Olivier B. (2008). Fear-potentiated startle and light-enhanced startle models in drug discovery. Curr. Protoc. Pharmacol..

[38] Hijzen T.H., Houtzager S.W., Joordens R.J., Olivier B., Slangen J.L. (1995). Predictive validity of the potentiated startle response as a behavioral model for anxiolytic drugs. Psychopharmacology.

[39] Barrenha G.D., Chester J.A. (2007). Genetic correlation between innate alcohol preference and fear-potentiated startle in selected mouse lines. Alcohol. Clin. Exp. Res..

[40] Barrenha G.D., Coon L.E., Chester J.A. (2011). Effects of alcohol on the acquisition and expression of fear-potentiated startle in mouse lines selectively bred for high and low alcohol preference. Psychopharmacology.

[41] Chester J.A., Kirchhoff A.M., Barrenha G.D. (2014). Relation between corticosterone and fear-related behavior in mice selectively bred for high or low alcohol preference. Addict. Biol..

[42] Crabbe J.C., Phillips T.J., Kosobud A., Belknap J.K. (1990). Estimation of genetic correlation: Interpretation of experiments using selectively bred and inbred animals. Alcohol Clin. Exp. Res..

[43] Powers M.S., Barrenha G.D., Mlinac N.S., Barker E.L., Chester J.A. (2010). Effects of the novel endocannabinoid uptake inhibitor, LY2183240, on fear-potentiated startle and alcohol-seeking behaviors in mice selectively bred for high alcohol preference. Psychopharmacology.

[44] Scherma M., Masia P., Satta V., Fratta W., Fadda P., Tanda G. (2019). Brain activity of anandamide: A rewarding bliss?. Acta Pharmacol. Sin..

[45] Arnold J.C., Topple A.N., Mallet P.E., Hunt G.E., McGregor I.S. (2001). The distribution of cannabinoid-induced Fos expression in rat brain: Differences between the Lewis and Wistar strain. Brain Res..

[46] Herkenham M., Lynn A.B., Johnson M.R., Melvin L.S., de Costa B.R., Rice K.C. (1991). Characterization and localization of cannabinoid receptors in rat brain: A quantitative in vitro autoradiographic study. J. Neurosci..

[47] Cippitelli A., Bilbao A., Hansson A.C., del Arco I., Sommer W., Heilig M., Massi M., Bermudez-Silva F.J., Navarro M., Ciccocioppo R. (2005). Cannabinoid CB1 receptor antagonism reduces conditioned reinstatement of ethanol-seeking behavior in rats. Eur. J. Neurosci..

[48] Oberlin B., Best C., Matson L., Henderson A., Grahame N. (2011). Derivation and characterization of replicate high- and low-alcohol preferring lines of mice and a high-drinking crossed HAP line. Behav. Genet..

[49] Paxinos G., Franklin K.B.J. (2001). The Mouse Brain in Stereotaxic Coordinates.

[50] Han B., Wright R., Kirchhoff A.M., Chester J.A., Cooper B.R., Davisson V.J., Barker E. (2013). Quantitative LC-MS/MS analysis of arachidonoyl amino acids in mouse brain with treatment of FAAH inhibitor. Anal. Biochem..

[51] Cooper R.L., Goldman J.M., Vandenbergh J.G., Chapin R.E., Heindel J.J. (1993). Monitoring of the estrous cycle in the laboratory rodent by vaginal lavage. Methods in Toxicology.

[52] Walker D.L., Davis M. (2002). Quantifying fear potentiated startle using absolute versus proportional increase scoring methods: Implications for the neurocircuitry of fear and anxiety. Psychopharmacology.

[53] Dixon W.J. (1950). Analysis of extreme values. Ann. Mathemat. Stat..

[54] Hill M.N., McLaughlin R.J., Morrish A.C., Viau V., Floresco S.B., Hillard C.J., Gorzalka B.B. (2009). Suppression of amygdalar endocannabinoid signaling by stress contributes to activation of the hypothalamic-pituitary-adrenal axis. Neuropsychopharmacology.

[55] Rademacher D.J., Meier S.E., Shi L., Ho W.S., Jarrahian A., Hillard C.J. (2008). Effects of acute and repeated restraint stress on endocannabinoid content in the amygdala, ventral striatum, and medial prefrontal cortex in mice. Neuropharmacology.

[56] Cohen H., Yehuda R. (2011). Gender differences in animal models of posttraumatic stress disorder. Dis. Markers.

[57] Mazor A., Matar M.A., Kaplan Z., Kozlovsky N., Zohar J., Cohen H. (2009). Gender-related qualitative differences in baseline and post-stress anxiety responses are not reflected in the incidence of criterion-based PTSD-like behaviour patterns. World J. Biol. Psychiatry.

[58] Ney L.J., Matthews A., Bruno R., Felmingham K.L. (2018). Modulation of the endocannabinoid system by sex hormones: Implications for posttraumatic stress disorder. Neurosci. Biobehav. Rev..

[59] Zer-Aviv T.M., Akirav I. (2016). Sex differences in hippocampal response to endocannabinoids after exposure to severe stress. Hippocampus.

[60] Sabatucci A., Simonetti M., Tortolani D., Angelucci C.B., Dainese E., Maccarrone M. (2019). Role of steroids on the membrane binding ability of fatty acid amide hydrolase. Cannabis Cannabinoid Res..

[61] Sumislawski J.J., Ramikie T.S., Patel S. (2011). Reversible gating of endocannabinoid plasticity in the amygdala by chronic stress: A potential role for monoacylglycerol lipase inhibition in the prevention of stress-induced behavioral adaptation. Neuropsychopharmacology.

[62] Morena M., Aukema R.J., Leitl K.D., Rashid A.J., Vecchiarelli H.A., Josselyn S.A., Hill M.N. (2019). Upregulation of anandamide hydrolysis in the basolateral complex of amygdala reduces fear memory expression and indices of stress and anxiety. J. Neurosci..

[63] Segev A., Korem N., Mizrachi Zer-Aviv T., Abush H., Lange R., Sauber G., Hillard C.J., Akirav I. (2018). Role of endocannabinoids in the hippocampus and amygdala in emotional memory and plasticity. Neuropsychopharmacology.

[64] Hill M.N., Tasker J.G. (2012). Endocannabinoid signaling, glucocorticoid-mediated negative feedback, and regulation of the hypothalamic-pituitary-adrenal axis. Neuroscience.

[65] de Oliveira Alvares L., Engelke D.S., Diehl F., Scheffer-Teixeira R., Haubrich J., de Freitas Cassini L., Molina V.A., Quillfeldt J.A. (2010). Stress response recruits the hippocampal endocannabinoid system for the modulation of fear memory. Learn. Mem..

[66] Lutz B., Marsicano G., Maldonado R., Hillard C.J. (2015). The endocannabinoid system in guarding against fear, anxiety and stress. Nat. Rev. Neurosci..

[67] Campolongo P., Roozendaal B., Trezza V., Hauer D., Schelling G., McGaugh J.L., Cuomo V. (2009). Endocannabinoids in the rat basolateral amygdala enhance memory consolidation and enable glucocorticoid modulation of memory. Proc. Natl. Acad. Sci. USA.

[68] Ganon-Elazar E., Akirav I. (2013). Cannabinoids and traumatic stress modulation of contextual fear extinction and GR expression in the amygdala-hippocampal-prefrontal circuit. Psychoneuroendocrinology.

[69] Gray J.M., Vecchiarelli H.A., Morena M., Lee T.T., Hermanson D.J., Kim A.B., McLaughlin R.J., Hassan K.I., Kuhne C., Wotjak C.T. (2015). Corticotropin-releasing hormone drives anandamide hydrolysis in the amygdala to promote anxiety. J. Neurosci..

[70] Hill M.N., McLaughlin R.J., Pan B., Fitzgerald M.L., Roberts C.J., Lee T.T., Karatsoreos I.N., Mackie K., Viau V., Pickel V.M. (2011). Recruitment of prefrontal cortical endocannabinoid signaling by glucocorticoids contributes to termination of the stress response. J. Neurosci..

[71] Bitencourt R.M., Pamplona F.A., Takahashi R.N. (2014). Corticosteroid-endocannabinoid loop supports decrease of fear-conditioned response in rats. Eur. Neuropsychopharmacol..

[72] Rey A.A., Purrio M., Viveros M.P., Lutz B. (2012). Biphasic effects of cannabinoids in anxiety responses: CB1 and GABA(B) receptors in the balance of GABAergic and glutamatergic neurotransmission. Neuropsychopharmacology.

[73] Tambaro S., Tomasi M.L., Bortolato M. (2013). Long-term CB(1) receptor blockade enhances vulnerability to anxiogenic-like effects of cannabinoids. Neuropharmacology.

[74] Franklin J.M., Mathew M., Carrasco G.A. (2013). Cannabinoid-induced upregulation of serotonin 2A receptors in the hypothalamic paraventricular nucleus and anxiety-like behaviors in rats. Neurosci. Lett..

[75] Caligioni C.S. (2009). Assessing reproductive status/stages in mice. Curr. Protoc. Neurosci..

[76] Gupta R.R., Sen S., Diepenhorst L.L., Rudick C.N., Maren S. (2001). Estrogen modulates sexually dimorphic contextual fear conditioning and hippocampal long-term potentiation (LTP) in rats. Brain Res..

[77] Zhao Y., Bijlsma E.Y., Verdouw M.P., Groenink L. (2018). No effect of sex and estrous cycle on the fear potentiated startle response in rats. Behav. Brain Res..

[78] Hiroi R., Neumaier J.F. (2006). Differential effects of ovarian steroids on anxiety versus fear as measured by open field test and fear-potentiated startle. Behav. Brain Res..

[79] Voulo M.E., Parsons R.G. (2019). Gonadal hormone fluctuations do not affect the expression or extinction of fear-potentiated startle in female rats. Behav. Neurosci..

